# Complex Interplay between HIV-1 Capsid and MX2-Independent Alpha Interferon-Induced Antiviral Factors

**DOI:** 10.1128/JVI.00458-16

**Published:** 2016-07-27

**Authors:** Lorenzo Bulli, Luis Apolonia, Juliane Kutzner, Darja Pollpeter, Caroline Goujon, Nikolas Herold, Sarah-Marie Schwarz, Yannick Giernat, Oliver T. Keppler, Michael H. Malim, Torsten Schaller

**Affiliations:** a Department of Infectious Diseases, Virology, Heidelberg University Hospital, Heidelberg, Germany; b Institute for Medical Virology, University Hospital Frankfurt/Main, Frankfurt, Germany; c Department of Infectious Diseases, King's College London, London, United Kingdom; d Childhood Cancer Research Unit, Astrid Lindgrens Children's Hospital, Karolinska Hospital, Stockholm, Sweden; University of Utah

## Abstract

Type I interferons (IFNs), including IFN-α, upregulate an array of IFN-stimulated genes (ISGs) and potently suppress Human immunodeficiency virus type 1 (HIV-1) infectivity in CD4^+^ T cells, monocyte-derived macrophages, and dendritic cells. Recently, we and others identified ISG myxovirus resistance 2 (MX2) as an inhibitor of HIV-1 nuclear entry. However, additional antiviral blocks exist upstream of nuclear import, but the ISGs that suppress infection, e.g., prior to (or during) reverse transcription, remain to be defined. We show here that the HIV-1 CA mutations N74D and A105T, both of which allow escape from inhibition by MX2 and the truncated version of cleavage and polyadenylation specific factor 6 (CPSF6), as well as the cyclophilin A (CypA)-binding loop mutation P90A, all increase sensitivity to IFN-α-mediated inhibition. Using clustered regularly interspaced short palindromic repeat (CRISPR)/Cas9 technology, we demonstrate that the IFN-α hypersensitivity of these mutants in THP-1 cells is independent of MX2 or CPSF6. As expected, CypA depletion had no additional effect on the behavior of the P90A mutant but modestly increased the IFN-α sensitivity of wild-type virus. Interestingly, the infectivity of wild-type or P90A virus could be rescued from the MX2-independent IFN-α-induced blocks in THP-1 cells by treatment with cyclosporine (Cs) or its nonimmunosuppressive analogue SDZ-NIM811, indicating that Cs-sensitive host cell cyclophilins other than CypA contribute to the activity of IFN-α-induced blocks. We propose that cellular interactions with incoming HIV-1 capsids help shield the virus from recognition by antiviral effector mechanisms. Thus, the CA protein is a fulcrum for the dynamic interplay between cell-encoded functions that inhibit or promote HIV-1 infection.

**IMPORTANCE** HIV-1 is the causative agent of AIDS. During acute HIV-1 infection, numerous proinflammatory cytokines are produced, including type I interferons (IFNs). IFNs can limit HIV-1 replication by inducing the expression of a set of antiviral genes that inhibit HIV-1 at multiple steps in its life cycle, including the postentry steps of reverse transcription and nuclear import. This is observed in cultured cell systems, as well as in clinical trials in HIV-1-infected patients. The identities of the cellular antiviral factors, their viral targets, and the underpinning mechanisms are largely unknown. We show here that the HIV-1 Capsid protein plays a central role in protecting the virus from IFN-induced inhibitors that block early postentry steps of infection. We further show that host cell cyclophilins play an important role in regulating these processes, thus highlighting the complex interplay between antiviral effector mechanisms and viral survival.

## INTRODUCTION

Acute human immunodeficiency virus type 1 (HIV-1) infection presents with a dramatic loss of CD4^+^ T cells, which is accompanied by the production of large quantities of cytokines (1, 2). Studies of simian immunodeficiency virus (SIV) infection of macaques suggest that this cytokine production contributes to initial limitation of viral spread, lowering the viral burden to a level defining the virological set point and facilitating the partial recovery of CD4^+^ T cell counts (3).

Type I interferons (IFNs), a group of cytokines released mainly by plasmacytoid dendritic cells during acute virus infection (4), include 13 different subtypes of IFN-α, as well as IFN-β, IFN-ε, IFN-κ, and IFN-ω (5), and have long been known to potently suppress HIV-1 replication in certain types of natural target cells (6–19). In addition to treating infections by other human pathogens (e.g., hepatitis C virus [HCV]), recombinant IFN-α therapy has also been investigated as a treatment strategy for HIV-1 infection. Although a substantial reduction in viral load was observed in chronic infection, viral rebound over time suggests that HIV-1 in-patient evolution may overcome IFN-α-induced antiviral host factors (20, 21). It is therefore likely that different HIV-1 strains have different sensitivities to type I IFNs. Comparison of diverse HIV-1 strains suggested that transmitted founder (T/F) viruses of subtype B, but not subtype C, show a relative resistance to IFN-α-induced blocks, arguing that type I IFNs may play an important role in limiting transmission in a subtype-defined context (22–24). The viral determinants for partially overcoming the IFN-α-induced blocks to HIV-1 are unknown. It is therefore important to identify the host cell effectors induced by type I IFNs and to understand the molecular interplay between the host and the virus after IFN-α treatment.

The addition of type I IFNs to cultured CD4^+^ T cells or monocyte-derived macrophages (MDMs) changes the expression profile of thousands of host genes (25) and induces the production of many antiviral proteins, only a few of which have been characterized in detail (reviewed in references 26 and 27). Preincubation of susceptible cells with type I IFNs blocks HIV-1 infection at an early step prior to or during reverse transcription (17, 28–31). The cellular host factors mediating this effect are unknown. One recently discovered type I IFN-induced factor that inhibits HIV-1 is the GTPase myxovirus resistance 2 (MX2 [also called MXB]) (32–34). MX2 blocks HIV-1 after reverse transcription at the level of nuclear entry, suggesting that IFN-induced host cell barriers are likely to interfere with HIV-1 infection at multiple early steps (32). Intriguingly, MX2 restriction of HIV-1 appears to be sensitive to changes in the HIV-1 Capsid protein (Gag^p24^/CA), an observation reminiscent of restriction by the bona fide CA binding factor TRIM5α (32–35). Consistent with these observations, *in vitro* studies suggest direct binding of MX2 to CA (36, 37). In addition, certain T/F viruses show some degree of resistance to the antiviral activity of MX2, suggesting a functional role of MX2 in limiting HIV-1 transmission (38).

The HIV-1 CA protein is essential for efficient infection and is genetically fragile, i.e., many amino acid residues are highly conserved and many single amino acid substitutions can abrogate viral infectivity (39, 40). Most likely, this is due to the central function of CA in the assembly and architecture of the viral core, a complex conical fullerene-like structure consisting of hexameric and pentameric CA subcomplexes (41–45). Amino acid substitutions in CA can affect capsid assembly, stability, and uncoating (46, 47), reverse transcription (48), usage of a productive nuclear import pathway (47, 49), and infection of nondividing cells (50), as well as integration site selection (51), processes that are all interlinked.

Some HIV-1 mutants carrying changes in CA, including N74D or P90A, have been shown to induce IFN-β secretion in MDMs or dendritic cells as a result of cyclic GMP-AMP synthase (cGAS)-mediated recognition of reverse transcription intermediates, indicating that the capsid shell may ordinarily shield viral DNA from recognition by innate immune sensors (52–54). The inability of these CA mutant viruses to replicate efficiently in MDMs (51, 55) may therefore be partially explained by a cascade of innate sensing, type I IFN secretion, and induction of IFN-stimulated genes (ISGs).

The HIV-1 CA mutant N74D escapes inhibition by a truncated version of cleavage and polyadenylation specific factor 6 (CPSF6-358) and does not bind a CPSF6-derived peptide *in vitro* (49, 56, 57). Similarly, CA mutant A105T is not restricted by CPSF6-358 (58). The cyclophilin-binding loop CA mutant P90A binds with reduced affinity to peptidylprolyl *cis-trans* isomerase A, also known as cyclophilin A (CypA), and is resistant to CypA-mediated isomerization of the G89-P90 peptide bond in CA (59–61). In addition to CypA (62, 63) and CPSF6 (56, 57), HIV-1 CA has been proposed to bind to the nuclear pore complex (NPC) protein NUP153 (64), as well as to the cyclophilin domain of NUP358 (51, 65), and binding-deficient CA mutants have been characterized. All of the aforementioned host proteins have been invoked as cofactors acting in early HIV-1 infection (49, 66–69).

While HIV-1 CA mutants can escape inhibition by ectopically expressed MX2, we now demonstrate that the infectivities of the HIV-1 CA mutants N74D, A105T, and P90A are hypersensitive to IFN-α-induced suppression compared to wild-type virus. This suggests that the susceptibility of viral determinants that are targeted by IFN-α-induced MX2-independent blocks is increased when capsid functionality is compromised. In addition, this MX2-independent IFN-α-induced postentry block can be relieved by pharmacological inhibition of cyclophilins, suggesting a role of host cell cyclophilins in the early type I IFN-induced suppression of HIV-1 infection. We suggest that the CA protein and the capsid core may therefore protect incoming HIV-1 nucleic acids from detection by innate pattern recognition receptors (52, 53), as well as IFN-α-induced effectors, thereby providing dual protection against host defense.

## MATERIALS AND METHODS

### Cells.

THP-1 cells were grown in RPMI 1640 GlutaMAX (Gibco) supplemented with 10% heat-inactivated fetal calf serum (FCS) and 100 U/ml penicillin and 100 μg/ml streptomycin. THP-1 cells were differentiated with 25 ng/ml phorbol 12-myristate 13-acetate (PMA; Sigma-Aldrich) for 24 h. The purification of primary blood mononuclear cells has been described before (70). Primary CD4^+^ T cells or CD14^+^ monocytes were derived from 50 ml of whole blood or from buffy coats and grown in RPMI 1640 medium with GlutaMAX with 10% heat-inactivated FCS and penicillin-streptomycin. CD4^+^ T cells were isolated using the CD4^+^ T cell isolation kit II (Miltenyi Biotec) or the RosetteSep human CD4^+^ T cell enrichment cocktail (Stem Cell Technologies), activated with 100 IU/ml interleukin-2 (IL-2; Biomol) and 2 μg/ml phytohemagglutinin (PHA; Sigma-Aldrich) for 3 days. MDMs were differentiated for 7 days using 100 ng/ml granulocyte-macrophage colony-stimulating factor (R&D Systems). 293T were grown in Dulbecco modified Eagle medium (DMEM-GlutaMAX; Gibco) with 10% heat-inactivated FCS and penicillin-streptomycin.

### Plasmids and viral vectors.

Vesicular stomatitis virus G protein (VSV-G)-pseudotyped wild-type- or CA mutant green fluorescent protein (GFP)-encoding HIV-1 vectors were produced using the Gag-Pol-encoding plasmid pCMV-ΔR8.91, the GFP reporter vector pCSGW, and the VSV-G-encoding plasmid pMD.G, which have been described before (51). pCMV-ΔR8.91-derived HIV-1 Gag-Pol plasmid encoding SIV_MAC_ CA has been described before (71). Full-length wild-type and CA mutant HIV-1 GFP reporter viruses were generated from pNLENG-IRES-Nef (51, 72). THP-1 clustered regularly interspaced short palindromic repeat (CRISPR)/Cas cells were generated by transduction with VSV-G-pseudotyped HIV-1 lentiviral particles produced using pCMV-ΔR8.91, pMD.G, and plentiCRISPRv2 (Addgene) (73, 74). Guide RNA encoding oligonucleotides (MWG/Eurofins) were annealed and cloned into BsmBI-linearized plentiCRISPRv2 according to the manufacturer's guidelines (Addgene). The oligonucleotides used were as follows (forward/reverse): for MX2g1, caccgAATTGACTTCTCCTCCGGTA/aaacTACCGGAGGAGAAGTCAATTc; for MX2g2, caccgACAAGCCTTGGCCCTACCGG/aaacCCGGTAGGGCCAAGGCTTGTc; for CPSF6g1, caccgATAGACATTTACGCGGATGT/aaacACATCCGCGTAAATGTCTATc; for CPSF6g2, caccgCATCCGCGTAAATGTCTATG/aaacCATAGACATTTACGCGGATGc; for CPSF6g3, caccgGGACCACATAGACATTTACG/aaacCGTAAATGTCTATGTGGTCCc; for CPSF6g4, caccgTCCATGTAATCTCGGTCTTC/aaacGAAGACCGAGATTACATGGAc; and for CypAg1, caccgGCCCGACCTCAAAGGAGACG/aaacCGTCTCCTTTGAGGTCGGGCc.

### Viral vector and HIV-1 production.

293T cells grown in 10-cm plates were transfected at a confluence of approximately 70 to 80% with 4.5 μg of viral vector plasmid, 3 μg of pCMVΔR8.91, and 3 μg of pMD.G using 4 μg of polyethylenimine per μg of DNA in 1 ml of Opti-MEM (Gibco) per plate. For VSV-G-pseudotyped full-length HIV-1 production, 8 μg of HIV-1 GFP reporter plasmid and 2 μg of pMD.G were cotransfected per plate. The medium was changed at 24 h posttransfection, the viruses were harvested at 48 and 72 h posttransfection and passed through a 0.45-μm-pore-size filter, and the collections were pooled. Depending on the experiment, viral supernatants were subjected to sucrose purification as described previously (70).

### Generation of CRISPR/Cas9 THP-1 cell clones.

THP-1 cells were transduced with VSV-G-pseudotyped HIV-1 lentiviral vector (LV) delivering plentiCRISPRv2 at an estimated multiplicity of infection (MOI) of 1. Transduced cell populations were selected with 1 μg/ml puromycin for 2 weeks. Single-cell clones were generated by limiting dilution and grown in 96-well plates for at least 2 weeks in the absence of puromycin. MX2 gene disruption was validated by PCR amplification of the targeted genomic region using the oligonucleotides AGCAAAGGAACATTGAGACTCTACTG (forward) and TTATTGTGGTGGGCTTACATGACAGC (reverse).

### Infections.

A total of 5 × 10^5^ THP-1 or CD4^+^ T cells were plated in 100 μl of medium per well in 96-well plates and treated for 24 h with IFN-α. Next, 100 μl of supernatant containing VSV-G-pseudotyped GFP-reporter lentiviral vectors or NL4.3GFP-reporter virus was added. To compare different CA mutants with wild-type GFP reporter vector/virus, we normalized viral input by 293T infectious titers or by units of reverse transcriptase in the supernatant, as determined by a SYBR green PCR-enhanced reverse transcriptase assay (SG-PERT) previously described (75). Cells were fixed in 4% paraformaldehyde (PFA) 2 to 3 days later, the infectivities were determined from the percentage of GFP^+^ cells by flow cytometry using a FACSVerse (BD Biosciences), and the infectious titers were determined on at least three different virus doses. The average infectious titers were calculated with standard deviations depicted as error bars. Experiments with MDMs were performed with 48-well plates, seeding 5 × 10^5^ monocytes per well prior to differentiation. For analysis by flow cytometry, MDMs were trypsinized for at least 30 min, resuspended, and fixed in 4% PFA. Virus titrations were usually performed by 3-fold serial dilutions of the viral supernatant. In the case of drug titration experiments (IFN-α or cyclosporine [Cs]), a single dose of supernatant containing reporter virus was used, aiming for an MOI of ≤1. For experiments using Cs or SDZ-NIM811, drugs were added at the time of infection with the reporter virus.

### Immunoblotting and antibodies.

Proteins were separated in Mini-Protean TGX stain-free precast gels (Bio-Rad) at 120 V for 1 h and subsequently subjected to UV activation and quantitative gel imaging to compare the amounts of loaded protein. Proteins were transferred to nitrocellulose membranes using Trans-Blot Turbo transfer system (Bio-Rad). The primary antibodies used were rabbit anti-MX1 (1:1,000; Proteintech), rabbit anti-MX2 (1:1,000; Novusbio), rabbit anti-CypA SA296 (1:3,000; Biomol), mouse anti-alpha-tubulin (1:3,000; Sigma-Aldrich), rabbit anti-CPSF6 (1:3,000; Abcam), and rabbit anti-MAPK (Erk1/2; 1:1,000; Cell Signaling). Goat anti-mouse or anti-rabbit IgG secondary antibodies were coupled to horseradish peroxidase (Cell Signaling Technology), and proteins were detected using Pierce ECL Plus Western blotting substrate (Thermo Scientific).

### Drugs.

SDZ-NIM811 was kindly provided by R. Bartenschlager (Heidelberg University Hospital, Heidelberg, Germany). Cyclosporine (Sandoz) was diluted in dimethyl sulfoxide (DMSO) to a stock of 1.0 mM. The IFN-α2 (Roferon-A, IFN-α-2a) stock concentration was 6.0 × 10^6^ IU/ml, and this was used at the indicated concentrations.

### TaqMan qPCR.

THP-1 cells were seeded at 10^6^ cells per well in six-well plates, treated or not with 500 IU/ml IFN-α for 24 h, and infected the next day with the same dose of wild-type or CA mutant viruses. The total DNA was isolated from the cells at 4 or 24 h postinfection using a QIAamp extraction kit (Qiagen). TaqMan quantitative PCR (qPCR) was performed using the GFP primers CAACAGCCACAACGTCTATATCAT/ATGTTGTGGCGGATCTTGAAG (forward/reverse) and the probe FAM-CCGACAAGCAGAAGAACGGCATCAA-TAMRA (where FAM is 6-carboxyfluorescein and TAMRA is 6-carboxytetramethylrhodamine) and the 2-long terminal repeat (2-LTR) circle primers AACTAGAGATCCCTCAGACCCTTTT/CTTGTCTTCGTTGGGAGTGAATT (forward/reverse) and the probe FAM-CTAGAGATTTTCCACACTGAC-TAMRA (76). The samples were normalized either to the total DNA concentration or by TaqMan qPCR for GAPDH (glyceraldehyde-3-phosphate dehydrogenase) with the primers GGCTGAGAACGGGAAGCTT/AGGGATCTCGCTCCTGGAA (forward/reverse) and the probe FAM-TCATCAATGGAAATCCCATCACCA-TAMRA. TaqMan qPCRs were performed using the Applied Biosystems 7500 real-time PCR system (Applied Biosystems).

## RESULTS

### HIV-1 CA mutants N74D or P90A display enhanced sensitivity to IFN-α-induced blocks.

**FIG 1:**
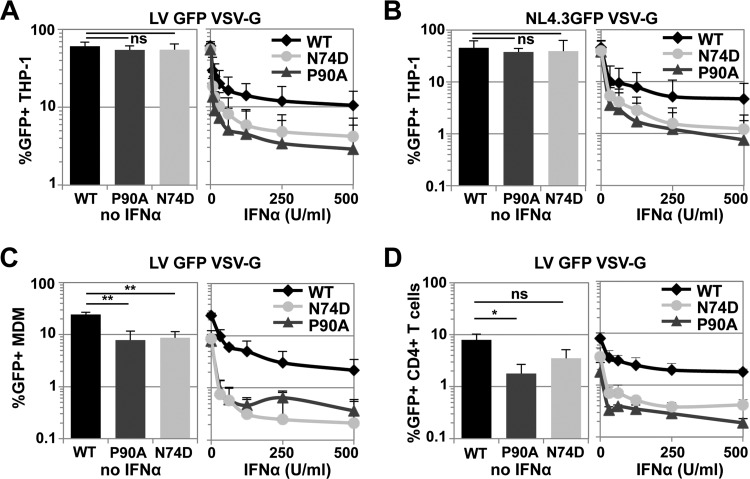
HIV-1 CA mutants N74D and P90A have increased sensitivity to IFN-α-induced suppression in diverse cell types. (A) THP-1 cells were pretreated with increasing doses of IFN-α and infected 24 h later with an equal amount of VSV-G-pseudotyped wild-type HIV-1 GFP lentiviral vector ΔR8.91 or CA mutant N74D or P90A, as measured by SG-PERT and 293T titration. At 48 h after infection the percentage of GFP-positive cells was determined by flow cytometry. Infectivities in the absence of IFN-α are shown on the left. The mean results from three independent experiments are shown. Error bars indicate the standard deviations. For untreated cells, a nonpaired two-tailed *t* test was performed (ns, not significant; *, *P* ≤ 0.05; **, *P* ≤ 0.005). For IFN-α-treated samples, a paired two-tailed *t* test with CI = 0.95 was performed (wild type [WT] versus P90A, *P* ≤ 0.0001; WT versus N74D, *P* ≤ 0.0001). (B) Same as for panel A but with NL4.3GFP-IRES-NEF (NL4.3GFP) viruses. Statistical evaluation was performed as in panel A (IFN-α-treated samples: WT versus P90A [*P* = 0.0012] and WT versus N74D [*P* = 0.0002]). (C) Same as for panel A, but with MDMs from three independent donors. Error bars indicate the standard deviations. The statistical tests were performed as in panel A (IFN-α-treated samples: WT versus P90A [*P* ≤ 0.0001] and WT versus N74D [*P* ≤ 0.0001]). (D) Same as for panel A, but with CD4^+^ T cells from three independent donors. Error bars indicate the standard deviations. Statistical analyses were analogous to panel A (IFN-α-treated samples: WT versus P90A [*P* ≤ 0.0001] and WT versus N74D [*P* ≤ 0.0001]).

Overexpression of the type I IFN-induced protein MX2 blocks HIV-1 infection in a CA-sensitive manner, and CA amino acid substitutions N74D or P90A reduce the sensitivity of HIV-1 to ectopic MX2-mediated repression (32, 33). We reasoned that N74D or P90A changes in HIV-1 CA might therefore reduce the sensitivity of HIV-1 to IFN-α-induced postentry blocks. To test this hypothesis, we treated the myeloid cell line THP-1 with increasing amounts of IFN-α and challenged with equal doses of wild-type VSV-G-pseudotyped HIV-1 GFP lentiviral vector (LV) or viruses with the CA mutants N74D or P90A, as judged by their infectious titers on 293T cells, and determined the percentages of infected cells 2 days later. In untreated THP-1 cells, no substantial differences in infectious titers were detected between the wild type or the CA mutants (Fig. 1A, left panel). Likewise, VSV-G-pseudotyped full-length NL4.3GFP reporter virus bearing wild-type CA or N74D or P90A mutant CA showed similar titers in THP-1 cells (Fig. 1B, left panel). In contrast, the CA mutants N74D or P90A were suppressed up to ~10-fold more efficiently by pretreatment with different concentrations of IFN-α (Fig. 1A and B, right panel).

We next examined these effects in MDMs or IL-2/PHA-activated CD4^+^ T cells. We confirmed that CA mutants N74D or P90A had modestly reduced infectivity in MDMs, as well as CD4^+^ T cells, compared to the wild type (51, 55, 69) (Fig. 1C and D, left panels). In both cell types, the N74D or P90A CA mutants were inhibited more effectively by pretreatment with lower doses of IFN-α relative to wild-type virus (Fig. 1C and D, right panels). For instance, in MDMs pretreated with 62.5 U of IFN-α/ml, N74D or P90A CA mutants were inhibited ∼20-fold, whereas wild-type HIV-1 GFP LV was blocked ∼4-fold (Fig. 1C). The hypersensitivity of HIV-1 CA mutants was also observed with NL4.3GFP carrying the Ba-L Envelope and thus was independent of the entry route (data not shown). In primary CD4^+^ T cells, CA mutants N74D or P90A were blocked ∼3-fold more than the wild-type virus at low IFN-α doses (Fig. 1D). These results reveal that HIV-1 CA mutants N74D or P90A have increased sensitivity to IFN-α-induced blocks: this was unanticipated given their reduced sensitivity to MX2-mediated inhibition (32). We therefore inferred that the IFN-α-induced factors blocking HIV-1 CA mutants N74D or P90A might function independently of MX2.

### The increased sensitivity of HIV-1 CA mutants to IFN-α-induced blocks is independent of MX2.

**FIG 2:**
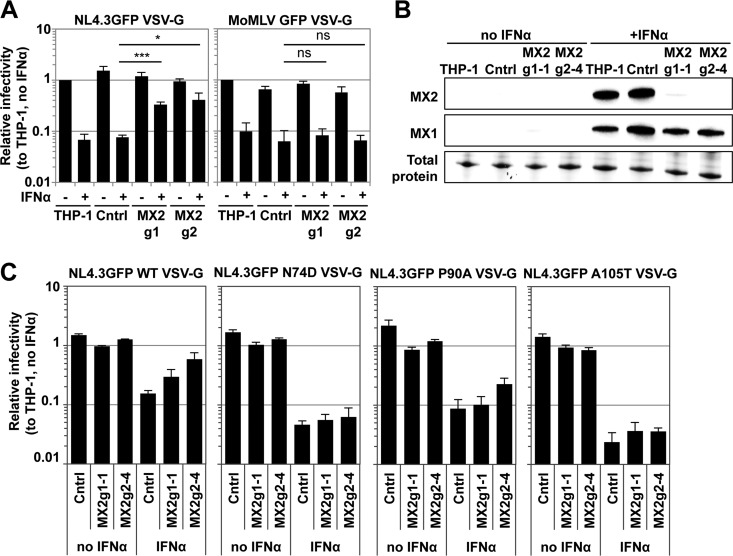
HIV-1 CA mutants are sensitized to MX2-independent IFN-α-induced blocks in THP-1 cells. (A) THP-1 cells were transduced with HIV-1 LVs encoding a control gRNA (Cntrl) or two independent gRNAs against MX2 (g1 and g2) and then puromycin selected for 2 weeks. Parental THP-1 cells or transduced CRISPR/Cas9 cell bulk populations were treated with 500 U/ml IFN-α and challenged 24 h later with a serial dilution of NL4.3GFP VSV-G reporter virus or Moloney murine leukemia virus GFP VSV-G vector. The percentages of infection were measured 2 days later by flow cytometry, and the relative infectivities were determined. Bars indicate the average infectious titers determined from at least three independent viral doses normalized to untreated THP-1 cells, and error bars indicate the standard deviations. Unpaired two-tailed *t* tests were performed (*, *P* ≤ 0.05; ***, *P* ≤ 0.001; ns, not significant). (B) Wild-type THP-1 cells, a CRISPR/Cas9 control, and two independent MX2 knockout THP-1 single cell clones (g1-1 and g2-4) were treated with 500 U/ml IFN-α2 for 24 h. Immunoblots of untreated or IFN-α-treated cells using antibodies specific for MX1 and MX2 are shown. Loading was controlled by measuring total protein by UV activation of the gel. (C) Wild-type THP-1 and the MX2 knockout cell clones (g1-1 and g2-4) were treated for 24 h with 500 U/ml IFN-α and then infected with a serial dilution of VSV-G-pseudotyped NL4.3GFP wild-type, CA N74D or CA P90A or CA A105T mutant viruses. The figure shows infection measured at 48 h as in panel A.

To demonstrate formally that the increased sensitivity of HIV-1 CA mutants N74D or P90A to IFN-α-induced blocks is independent of MX2, we disrupted the MX2 gene in THP-1 cells using two independent CRISPR guide RNAs (MX2g1 and MX2g2). These guide RNAs target exon 1 immediately after the start ATG, thereby disrupting expression of the 715-amino-acid full-length MX2 protein that possesses antiviral activity (the expression of a slightly shorter nonantiviral isoform initiating from a downstream ATG may still be possible). Transduced THP-1 bulk populations expressing either of the two guide RNAs displayed an approximately 5- to 8-fold reduced block to HIV-1 NL4.3GFP VSV-G infection after IFN-α treatment (Fig. 2A). As controls, we used parental THP-1 cells, as well as the CRISPR/Cas9 control (Cntrl) cells, which had similar permissivity to HIV-1 infection and expressed comparable MX1 and MX2 levels after IFN-α stimulation (Fig. 2A and B). In contrast, a Moloney murine leukemia virus GFP vector, which is unaffected by ectopic MX2 overexpression (32), was inhibited by IFN-α in control cells and MX2 CRISPR/Cas9 cells to similar extents (Fig. 2A).

Subsequently, single cell clones were derived in which MX2 expression after IFN-α stimulation was either ablated (MX2g2-4) or substantially reduced (MX2g1-1), whereas the IFN-α signaling pathway remained intact, as measured by MX1 induction (Fig. 2B). We validated gene disruption by PCR amplifying the targeted genomic region and found that MX2g1-1 harbored two heterozygous deletions, whereas MX2g2-4 had a homozygous deletion, in both cases leading to disruption of the MX2 open reading frame (data not shown). MX2g1-1, as well as MX2g2-4 and Cntrl THP-1 cells, showed no substantial difference in infection by wild-type HIV-1 GFP reporter virus or CA mutants N74D or P90A in the absence of IFN-α prestimulation (Fig. 2C). In IFN-α-stimulated THP-1 cells, infectivity of wild-type virus decreased ∼10-fold, whereas in MX2g1-1 and MX2g2-4 cells the infectivity was (as expected) reduced only approximately 3- to 5-fold (Fig. 2C). In contrast, infectivity of the N74D mutant decreased in IFN-α-stimulated parental THP-1 cells ∼43-fold and in MX2g1-1 and MX2g2-4 cells ∼20-fold (Fig. 2C). Similarly, HIV-1 P90A was inhibited more strongly by IFN-α treatment in parental THP-1, as well as MX2 CRISPR/Cas9 cells, relative to the wild-type virus (Fig. 2C). These data demonstrate that both HIV-1 CA N74D and P90A are hypersensitive to MX2-independent IFN-α-induced antiviral effectors.

HIV-1 N74D does not bind to the cleavage and polyadenylation specific factor 6 (CPSF6) and is insensitive to an artificially truncated version of this protein (CPSF6-358) which blocks wild-type HIV-1 infection efficiently (49, 56, 57). To test whether a lack in CPSF6 binding to CA could explain the enhanced sensitivity of HIV-1 CA N74D mutant to IFN-α-induced blocks, we studied HIV-1 CA A105T, another CPSF6-independent CA mutant. Like N74D or P90A, HIV-1 A105T was less sensitive to inhibition by ectopically expressed MX2 (data not shown). The A105T mutant was inhibited ∼50-fold in THP-1 cells after IFN-α pretreatment, but like N74D, was not efficiently rescued from this block in cells lacking MX2 (Fig. 2C). We also observed increased sensitivity of N74D or A105T viruses to IFN-α-induced blocks in PMA-differentiated MX2 CRISPR/Cas9 THP-1 cells, as well as in MDMs, indicating that these blocks function independently of cell proliferation (data not shown). Together, these data raised the possibility that the interaction of incoming HIV-1 CA with CPSF6 may help protect infection from MX2-independent IFN-α-induced blocks.

### CPSF6 does not contribute to the IFN-α-induced HIV-1 blocks.

**FIG 3:**
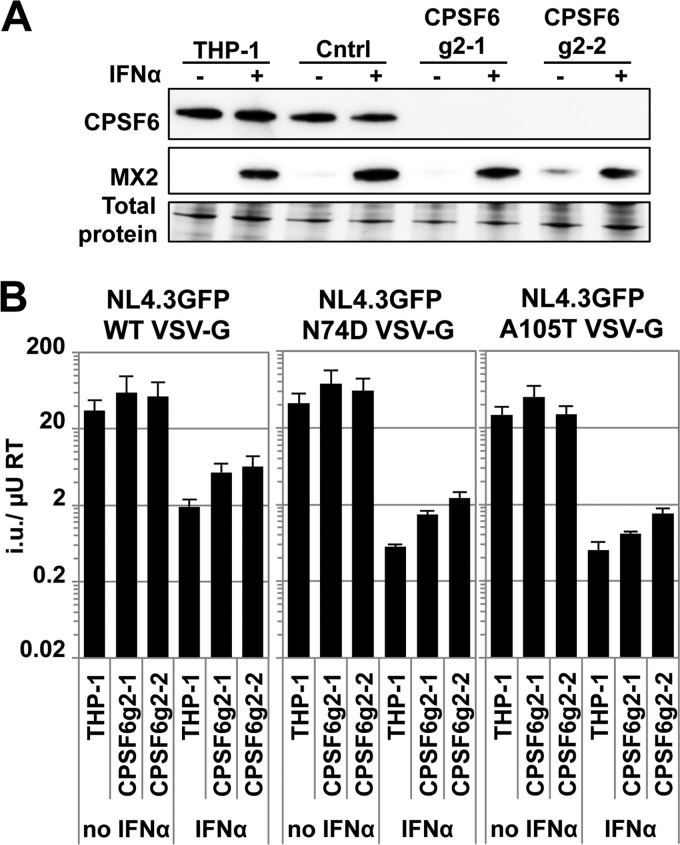
CPSF6 knockout in THP-1 cells does not increase sensitivity to IFN-α-induced blocks to HIV-1. (A) Parental THP-1 cells or three independent CRISPR/Cas9 cell clones of which two did not express CPSF6 (g2-1 and g2-2) were treated 24 h with IFN-α or left untreated. The next day, whole-cell lysates were used in immunoblotting experiments to detect CPSF6 expression levels. MX2 expression served as a control for IFN-α treatment. (B) In parallel, cells were infected 24 h after IFN-α treatment with a serial dilution of VSV-G pseudotyped wild-type NL4.3GFP or the CA mutants N74D or A105T. The average infectious titers (i.u./μU RT) of three virus doses are shown, and error bars indicate the standard deviations.

To address the possible role of CPSF6 in protecting HIV-1 from IFN-α-induced antiviral factors, we sought to determine whether the sensitivity to IFN-α was increased when CPSF6 protein levels were reduced. We therefore generated THP-1 CPSF6 CRISPR/Cas9 cells using four independent guide RNAs (CPSF6g1 to CPSF6g4). In stably transduced and drug selected bulk cultures expressing CPSF6 gRNAs, IFN-α treatment reduced HIV-1 GFP reporter virus infectivity to the same extent as in parental cells (∼20-fold, data not shown). We next generated single cell clones and proceeded with two lines with no CPSF6 expression (CPSF6g2-1 and CPSF6g2-2) and one control clone (Cntrl) in which CPSF6 expression matched that of parental cells, as well as parental THP-1 (Fig. 3A). CPSF6 levels did not change upon IFN-α treatment in any of the cell clones (Fig. 3A). Wild-type and N74D or A105T viruses showed similar titers in the untreated parental, as well as CPSF6-depleted cell clones, and as expected, the N74D or A105T viruses showed increased sensitivity to IFN-α pretreatment in all cells (Fig. 3B). Notably, the sensitivity of wild-type HIV-1 to IFN-α treatment was not increased in the CPSF6-depleted cells, supporting a model in which CPSF6 binding to incoming HIV-1 cores does not confer protection from type I IFN-induced effectors (Fig. 3B). The increased sensitivity of N74D or A105T viruses to IFN-α therefore cannot be explained by a lack of CPSF6 binding. Unexpectedly, infection with all three viruses was mildly increased in CPSF6g2-1 and CPSF6g2-2 cells (Fig. 3B). Given its cellular role, it is possible that the lack of CPSF6 perturbs the mRNA processing and expression of ISGs involved in inhibiting HIV-1.

### IFN-α induces an enhanced block to reverse transcription and nuclear import of HIV-1 CA N74D.

**FIG 4:**
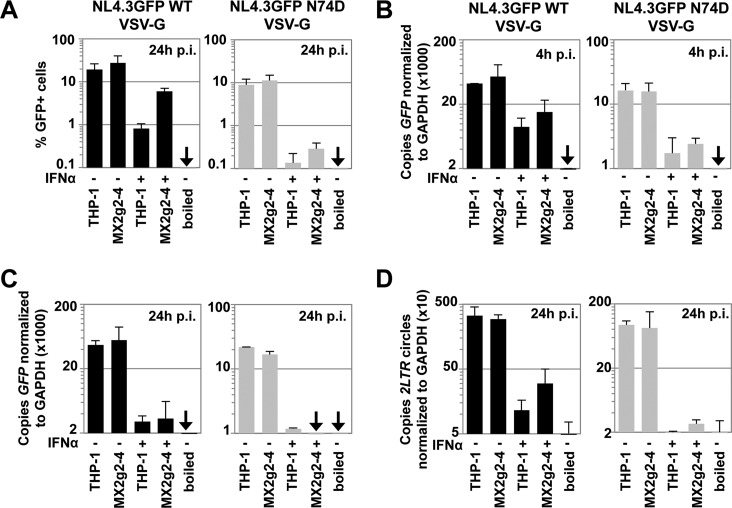
Hypersensitivity of the HIV-1 CA mutant N74D to IFN-α occurs at the stages of reverse transcription and nuclear import. Parental THP-1 or MX2g2-4 CRISPR/Cas9 cells were infected with VSV-G-pseudotyped NL4.3GFP wild-type or N74D CA mutant virus. (A) At 24 h after infection, the percentages of GFP-positive cells were determined by flow cytometry. (B to D) In two parallel samples, total DNA was extracted 4 h (B) or 24 h (C) postinfection and subjected to TaqMan quantitative PCR using a primer/probe set specific for *GFP* or 2-LTR circles (D). As a control for plasmid contamination, boiled virus was used. The averages of two independent experiments are shown.

We next investigated the stage(s) of infection that is blocked in MX2-depleted cells. We measured the infectivities of VSV-G-pseudotyped wild-type NL4.3GFP or the N74D mutant in THP-1 or CRISPR/Cas9 MX2g2-4 cells (Fig. 4A) and in parallel isolated total DNA 4 or 24 h after infection to measure the amounts of *GFP* reverse transcription product (Fig. 4B and C) or 2-LTR circles (Fig. 4D), respectively, by TaqMan quantitative PCR. As controls for plasmid contamination, we used viral supernatants that had been inactivated by boiling. The levels of GFP reverse transcription products from HIV-1 wild-type virus decreased after IFN-α pretreatment ∼4-fold at 4 h postinfection (Fig. 4B). In contrast, the levels of GFP reverse transcription products for HIV-1 CA N74D were reduced after IFN-α treatment ∼8-fold and were not substantially different in MX2g2-4 cells compared to parental THP-1 (Fig. 4B). We also observed a strong reduction in copy numbers of *GFP* reverse transcription products 24 h postinfection for both the wild type and the N74D mutant (Fig. 4C). In a parallel sample, we analyzed 2-LTR circles, a surrogate for HIV-1 nuclear import, at 24 h postinfection. We found that after IFN-α pretreatment and wild-type HIV-1 infection, 2-LTR circles were reduced in parental THP-1 cells ∼30-fold, and only ~10-fold in MX2g2-4 cells, corroborating the role for MX2 in blocking nuclear import (Fig. 4D). While for wild-type virus the 2-LTR circles were reduced ∼9-fold in IFN-α-treated MX2g2-4 cells, the 2-LTR circles for N74D were blocked by almost 2 orders of magnitude (Fig. 4D). Therefore, the increased IFN-α-induced block to N74D infection (Fig. 4A) can be explained by a stronger reduction in reverse transcription products (Fig. 4B and C) and reduced accumulation of 2-LTR circles, reflecting the suppression of nuclear import (Fig. 4D). In conclusion, our data suggest that wild-type CA protects HIV-1 from MX2-independent IFN-α-induced factors that target reverse transcription and nuclear import and that the CA changes increase sensitivity to inhibition by these factors.

### Host cell cyclophilins regulate IFN-α-induced blocks to HIV-1.

**FIG 5:**
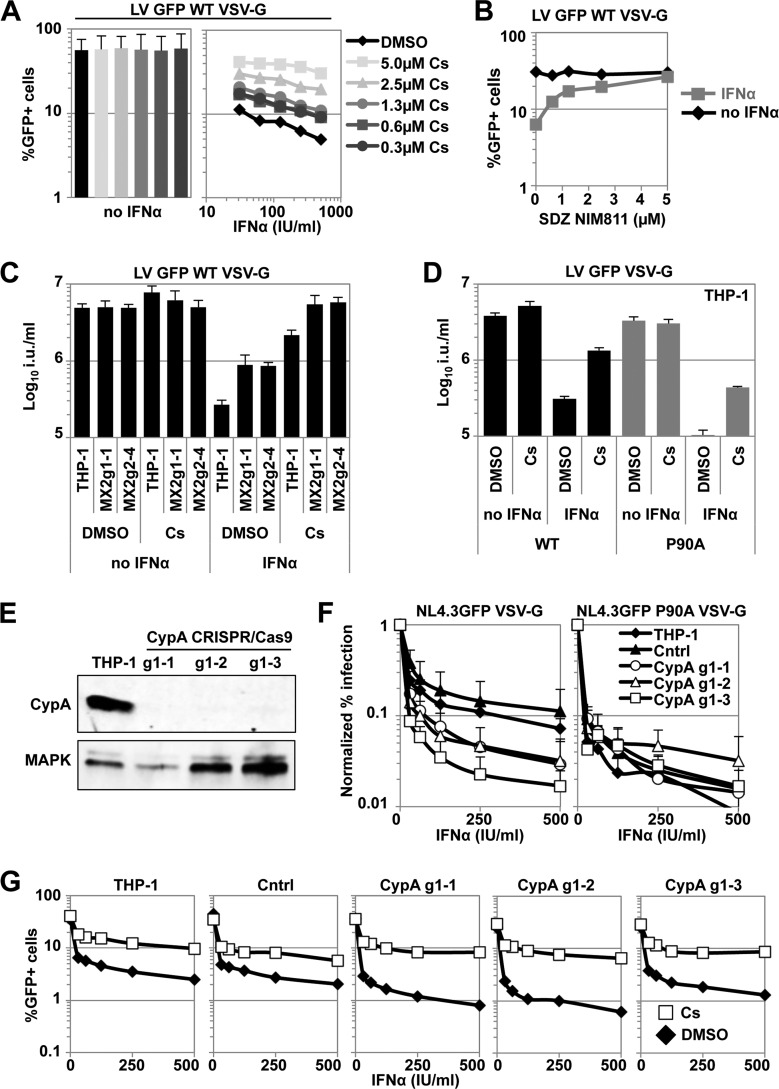
Cyclophilins affect the sensitivity of HIV-1 to IFN-α-induced effectors in THP-1 cells. (A) THP-1 cells were treated for 24 h with a serial dilution of IFN-α before infection with VSV-G-pseudotyped wild-type HIV-1 GFP vector (ΔR8.91) in the presence of increasing amounts of Cs in DMSO. The percentages of infected cells were measured 48 h later by flow cytometry. Representative results from three independent experiments are shown. (B) THP-1 cells were treated for 24 h with 100 U/ml IFN-α or left untreated and then infected with a single dose of VSV-G-pseudotyped wild-type HIV-1 GFP LV in the presence of increasing amounts of the cyclosporine analogue SDZ-NIM811. The percentages of infected cells were determined as in panel A. Representative results from three independent experiments are shown. (C) Parental THP-1 cells or MX2 CRISPR/Cas9 cell clones were pretreated with 500 U/ml IFN-α for 24 h and then infected with a serial dilution of VSV-G-pseudotyped wild-type HIV-1 GFP LV in the presence or absence of 5 μM Cs or DMSO as a control, and the percentages of GFP-positive cells were determined as in panel A. Average infectious titers for three viral doses are shown. Error bars indicate the standard deviations. (D) THP-1 cells were treated for 24 h with 500 U/ml IFN-α and then infected with a serial dilution of VSV-G-pseudotyped wild-type or CA P90A mutant HIV-1 GFP LV in the presence or absence of 2.5 μM Cs or DMSO as a carrier control. The percentages of infected cells were determined as in panel A. (E) THP-1 cells were transduced with a vector expressing a CRISPR/Cas9 guide RNA against CypA, and individual single cell clones were tested. Immunoblotting of cell lysates from parental THP-1 as well as three independent THP-1 CypA CRISPR/Cas9 cell clones detecting CypA and with MAPK as a loading control. (F) Parental THP-1 cells, Cntrl cells, or CypA CRISPR/Cas9 cell clones were treated with a serial dilution of IFN-α for 24 h and then infected with VSV-G-pseudotyped wild-type or CA P90A mutant NL4.3GFP at similar doses. The percentages of infected cells were determined as for panel A, and normalized infectivities were determined in the absence of IFN-α treatment. The average relative infectivities of three independent experiments are shown. Error bars indicate the standard deviations. For data derived from IFN-α-treated samples, a paired two-tailed *t* test with confidence interval of 0.95 was performed (HIV-1 NL4.3GFP wild type: Cntrl versus CypAg1-1 [**, *P* = 0.0047]; Cntrl versus CypAg1-2 [**, *P* = 0.0058]; Cntrl versus CypAg1-3 [***, *P* = 0.0002]; NL4.3GFP P90A: Cntrl versus CypAg1-1 [n.s.]; Cntrl versus CypAg1-2 [n.s.]; Cntrl versus CypAg1-3 [n.s.]; Cntrl cells: NL4.3GFP wild type versus NL4.3GFP P90A [***, *P* < 0.0001]). Without IFN-α treatment, no significant differences between Cntrl cells and CypAg1-1, CypAg1-2, or CypAg1-3 were detected for both NL4.3GFP wild type and NL4.3GFP P90A. (G) Parental THP-1 cells, Cntrl cells, or CypA CRISPR/Cas9 cell clones were treated with a serial dilution of IFN-α for 24 h and then infected with VSV-G-pseudotyped HIV-1 GFP LV in the presence of 5 μM DMSO or Cs, and the percentages of infected cells were determined as for panel A. Representative results from three independent experiments are shown.

Since we observed an increased sensitivity of the P90A CA mutant virus to IFN-α-induced MX2-independent antiviral effectors (Fig. 1), we hypothesized that the host protein cyclophilin A (CypA), which can isomerize the G89-P90 peptide bond in HIV-1 CA, might affect IFN-α-induced blocks. CypA is a target for the immunosuppressive drug cyclosporine (Cs), as well as for nonimmunosuppressive compounds such as SDZ-NIM811. In the presence of Cs or SDZ-NIM811, virus infectivity is often reduced, suggesting that CypA and possibly other cyclophilins may act as cofactors that promote HIV-1 infection (59, 69, 77–80). We treated THP-1 cells with serial dilutions of IFN-α for 24 h, challenged them with VSV-G-pseudotyped HIV-1 GFP LV in the presence of increasing amounts of Cs, and determined the levels of infection at 2 days postinfection. Unexpectedly, we found that Cs addition rescued HIV-1 LV infectivity in a dose-dependent manner, e.g., the infectivity was increased ∼10-fold with 5 μM Cs (Fig. 5A). To rule out an immunosuppressive effect mediated through calcineurin (81), we analyzed whether the nonimmunosuppressive Cs analogue SDZ-NIM811 (77) would also rescue infectivity. We treated THP-1 cells for 24 h with IFN-α and then infected them with a single dose of HIV-1 GFP LV in the presence of increasing doses of SDZ-NIM811 up to 5 μM. Similar to Cs, SDZ-NIM811 rescued the infectivity of wild-type HIV-1 LV from the IFN-α-induced block (Fig. 5B), suggesting that the reversion of IFN-α-induced inhibition of HIV-1 infection was independent of the immunosuppressive activity of Cs.

The reduction of CypA levels or pharmacologic inhibition by the provision of Cs, as well as CA manipulation to escape CypA binding, has been shown to overcome the antiviral effect of MX2 (34). The efficient rescue of HIV-1 GFP LV infectivity from the IFN-α-induced blocks in THP-1 cells by Cs (Fig. 5A) suggested that Cs could affect both the block mediated through MX2 and the MX2-independent IFN-α-induced blocks. To investigate this, we incubated parental THP-1 cells or the MX2-deficient lines MX2g1-1 or MX2g2-4 with IFN-α for 24 h and challenged them with VSV-G-pseudotyped HIV-1 GFP LV in the presence or absence of Cs. We observed a rescue of virus infectivity in THP-1 cells and in MX2-deficient cell clones by Cs (Fig. 5C). Surprisingly, when we tested VSV-G-pseudotyped HIV-1 GFP LV P90A in IFN-α-stimulated cells, we also observed an increased infectivity in the presence of Cs (Fig. 5D). These data demonstrate that the MX2-independent IFN-α-induced blocks are sensitive to Cs.

Cs does not exclusively target CypA but can also bind and inhibit other cyclophilins. Therefore, we decided to investigate whether *CypA* gene disruption would either increase the sensitivity of wild-type HIV-1 to IFN-α, phenocopying the P90A mutant, or would rescue wild type virus infectivity from the IFN-α-induced block, as observed with Cs treatment. We generated THP-1 cell clones in which *CypA* was disrupted using CRISPR/Cas9 technology. CypA expression was eliminated in three different clones, as judged by immunoblotting (Fig. 5E). These stable cell clones were pretreated with a serial dilution of IFN-α for 24 h and challenged with VSV-G-pseudotyped NL4.3GFP or the corresponding CA mutant virus NL4.3GFP P90A. *CypA* gene disruption had no major effects on the infectivities of wild-type or P90A mutant virus in the absence of IFN-α stimulation. In contrast, after IFN-α treatment, the infectivity of wild-type virus was reduced ∼5-fold more in THP-1 cells lacking CypA than that in parental or Cntrl cells (Fig. 5F). The infectivity of the P90A mutant virus was reduced by IFN-α to similar levels in all cell lines, suggesting that the P90A mutant phenocopied wild-type infections in the absence of CypA (Fig. 5E and F).

Because these data indicated that CypA gene disruption and Cs treatment had opposing effects on the infectivity of HIV-1 in the presence of IFN-α, we needed to rule out a role of CypA in the Cs-mediated increase of HIV-1 infectivity. We stimulated the THP-1 cell clones lacking CypA with a serial dilution of IFN-α and challenged with wild-type HIV-1 GFP LV in the presence or absence of Cs. Strikingly, Cs also rescued HIV-1 infectivity from the IFN-α-induced block in cells lacking CypA (Fig. 5G). Collectively, these findings indicate that whereas CypA helps protect incoming HIV-1 cores from IFN-α-induced antiviral factors, the activity of other cyclophilins contributes substantially to the IFN-α-induced block in THP-1 cells.

## DISCUSSION

HIV-1 infection is blocked during reverse transcription by IFN-α pretreatment of certain cell types, including CD4^+^ T cells and MDMs (30). The physiological importance of type I IFNs during the early course of HIV-1 infection is evidenced by the observation that certain T/F viruses replicate more efficiently in the presence of IFN-α than in their viral counterparts from chronic infection (22, 23). Likewise, the plasma SIV viral load in acutely infected macaques was increased by the addition of a type I IFN receptor antagonist, arguing for an important functional role of type I IFN in suppressing viral replication during the acute phase (3). In addition, clinical trials have demonstrated reductions in viral load during IFN-α treatment, making therapy strategies incorporating IFN-α treatment attractive (82, 83). However, plasma HIV-1 RNA rebounds to a degree after several weeks, suggesting HIV-1 escape from, or desensitization to, IFN-α-induced antiviral effectors (83). The viral determinants for the differential sensitivity to type I IFN-induced blocks are currently unknown.

Here, we analyzed the sensitivity of HIV-1 CA mutants N74D, A105T, as well as P90A, to IFN-α-induced blocks and found that their infectivities were reduced further after IFN-α treatment of THP-1 cells, MDMs, or CD4^+^ T cells than that of wild-type virus (Fig. 1), despite their relative resistance to ectopically expressed MX2 (32–34). Using genetic knockout, the increased sensitivity of the HIV-1 CA mutants to IFN-α-induced blocks was shown to be independent of MX2 (Fig. 2) and occurred during reverse transcription (Fig. 4), suggesting that alterations in CA may lead to enhanced interactions between IFN-α-induced antiviral effectors and CA (perhaps as a consequence of slower capsid uncoating [84]) and/or to detrimental exposure of reverse transcription complexes to such effectors. During the preparation of the manuscript, Opp et al. reported that MX2 gene disruption had no restorative effect on HIV-1 infectivity from an IFN-α-induced block in THP-1 cells (85). Our results differ substantially and confirm that IFN-α induces at least two blocks, the first one at the level of reverse transcription (30), which is sensitive to changes in CA, and the second one at the level of nuclear import and involving MX2 (32).

Disruption of *CypA* by CRISPR/Cas9 modestly increased the sensitivity of HIV-1 to IFN-α-induced blocks, indicating that CypA interactions with incoming capsids may help to protect HIV-1 from IFN-α-induced antiviral effectors in THP-1 cells (Fig. 5). In contrast, genetic disruption of CPSF6 did not increase the sensitivity of wild-type or CA mutant HIV-1 to IFN-α-induced effectors in THP-1 cells (Fig. 3), arguing that CPSF6 does not play a role in protecting infection from IFN-α-induced blocks (49, 58). The findings therefore differ from what has been described for cGAS sensing of HIV-1 where both CypA and CPSF6 are thought to play protective roles (53). However, the replication defects of N74D and P90A viruses in MDMs may be explained by a combination of increased cGAS sensing, IFN-α induction, and increased sensitivity to the IFN-α-induced effectors themselves.

Our data suggest that at least one other Cs-sensitive cyclophilin, other than CypA, is directly or indirectly involved in the IFN-α-induced early block to HIV-1. Other cyclophilins for which evidence suggests interactions with HIV-1 proteins include PPIB (62), PPIE and PPIF (86), and PPIH (87). The nuclear pore complex component NUP358, a cofactor for HIV-1 early infection steps, has a cyclophilin domain at its carboxy terminus; however, this domain does not efficiently interact with Cs, making it unlikely that NUP358 is involved in the Cs/SDZ-NIM811-mediated rescue of infectivity from IFN-α-induced blocks (51, 65). Screening the interferome database (88) revealed that human peptidylprolyl isomerase F (PPIF) transcripts were upregulated 2-fold when CD14^+^ monocytes were treated with 10,000 IU/ml IFN-α (data set IFM30) and mouse Nktr and Ranbp2 were upregulated 2.6- and 3.4-fold, respectively, by 10,000 IU/ml IFN-β (data set IFM34). To our knowledge, these are the only cyclophilins that have been suggested to be upregulated by type I IFNs. Importantly, Cs addition also rescued HIV-1 P90A infectivity with an efficiency similar to that of the wild type (Fig. 5D), arguing that the G89-P90 peptide bond in the cyclophilin-binding loop of CA is not the responsive viral determinant.

Multiple lines of evidence suggest that CA mutants N74D, A105T or P90A have defects in capsid stability and uncoating, possibly due to altered interactions with host factors. First, cDNA reverse transcription intermediates of both N74D and P90A mutant viruses are sensed and trigger type I IFN production after infection of MDMs (53). Second, N74D shows delayed uncoating kinetics in TRIMCyp-expressing cells and a Cs washout assay (89), suggesting that this mutation perturbs uncoating (84). Third, N74D is more susceptible to the NNRTI nevirapine compared to wild-type virus, suggesting that wild-type capsid integrity may help protect the virus from antiviral drugs (55). Fourth, HIV-1 CA P90A binds CypA with reduced affinity (51, 60), such that the stabilizing effects of CypA binding to CA are lost (90, 91). Fifth, the A105T mutation, though outside the cyclophilin-binding loop, can affect viral sensitivity to Cs (92–95). Finally, all three CA mutants change the requirement for specific NPC-associated proteins during infection, suggesting that events before reaching the NPC are altered (49). Whether cytoplasmic trafficking is disturbed for HIV-1 CA mutants is unclear at the moment (96). Accordingly, interactions with cellular factors, as well as the intrinsic properties of incoming viral capsids, are affected by these particular mutations, one consequence of which is the greater sensitivity of relevant mutant viruses to IFN-α-induced infectivity blocks.
